# The plasma peptides of sepsis

**DOI:** 10.1186/s12014-020-09288-5

**Published:** 2020-07-02

**Authors:** Thanusi Thavarajah, Claudia C. dos Santos, Arthur S. Slutsky, John C. Marshall, Pete Bowden, Alexander Romaschin, John G. Marshall

**Affiliations:** 1 Ryerson Analytical Biochemistry Laboratory (RABL), Department of Chemistry and Biology, Faculty of Science, Ryerson University, 350 Victoria St., Toronto, ON Canada; 2 St. Michael’s Hospital, Keenan Research Centre for Biomedical Science, Toronto, Canada; 3 St. Michael’s Hospital, Keenan Chair in Medicine, University of Toronto, Toronto, Canada; 4 International Biobank of Luxembourg (IBBL), Institute of Health (formerly CRP Sante Luxembourg), Dudelange, Luxembourg

**Keywords:** Human EDTA plasma, Organic extraction, Nano chromatography, Electrospray ionization tandem mass spectrometry, LC–ESI–MS/MS, Linear quadrupole ion trap, Discovery of variation, Intensive Care Unit, Sepsis, Random and independent sampling, Chi square test and ANOVA, SQL SERVER & R

## Abstract

**Background:**

A practical strategy to discover sepsis specific proteins may be to compare the plasma peptides and proteins from patients in the intensive care unit with and without sepsis. The aim was to discover proteins and/or peptides that show greater observation frequency and/or precursor intensity in sepsis. The endogenous tryptic peptides of ICU-Sepsis were compared to ICU Control, ovarian cancer, breast cancer, female normal, sepsis, heart attack, Alzheimer’s and multiple sclerosis along with their institution-matched controls, female normals and normal samples collected directly onto ice.

**Methods:**

Endogenous tryptic peptides were extracted from individual sepsis and control EDTA plasma samples in a step gradient of acetonitrile for random and independent sampling by LC–ESI–MS/MS with a set of robust and sensitive linear quadrupole ion traps. The MS/MS spectra were fit to fully tryptic peptides within proteins using the X!TANDEM algorithm. The protein observation frequency was counted using the SEQUEST algorithm after selecting the single best charge state and peptide sequence for each MS/MS spectra. The protein observation frequency of ICU-sepsis versus ICU Control was subsequently tested by Chi square analysis. The average protein or peptide log_10_ precursor intensity was compared across disease and control treatments by ANOVA in the R statistical system.

**Results:**

Peptides and/or phosphopeptides of common plasma proteins such as ITIH3, SAA2, SAA1, and FN1 showed increased observation frequency by Chi square (χ^2^ > 9, p < 0.003) and/or precursor intensity in sepsis. Cellular gene symbols with large Chi square values from tryptic peptides included POTEB, CTNNA1, U2SURP, KIF24, NLGN2, KSR1, GTF2H1, KIT, RPS6KL1, VAV2, HSPA7, SMC2, TCEB3B, ZNF300, SUPV3L1, ADAMTS20, LAMB4, MCCC1, SUPT6H, SCN9A, SBNO1, EPHA1, ABLIM2, cB5E3.2, EPHA10, GRIN2B, HIVEP2, CCL16, TKT, LRP2 and TMF1 amongst others showed increased observation frequency. Similarly, increased frequency of tryptic phosphopeptides were observed from POM121C, SCN8A, TMED8, NSUN7, SLX4, MADD, DNLZ, PDE3B, UTY, DEPDC7, MTX1, MYO1E, RXRB, SYDE1, FN1, PUS7L, FYCO1, USP26, ACAP2, AHI1, KSR2, LMAN1, ZNF280D and SLC8A2 amongst others. Increases in mean precursor intensity in peptides from common plasma proteins such as ITIH3, SAA2, SAA1, and FN1 as well as cellular proteins such as COL24A1, POTEB, KANK1, SDCBP2, DNAH11, ADAMTS7, MLLT1, TTC21A, TSHR, SLX4, MTCH1, and PUS7L among others were associated with sepsis. The processing of SAA1 included the cleavage of the terminal peptide D/PNHFRPAGLPEKY from the most hydrophilic point of SAA1 on the COOH side of the cystatin C binding that was most apparent in ICU-Sepsis patients compared to all other diseases and controls. Additional cleavage of SAA1 on the NH2 terminus side of the cystatin binding site were observed in ICU-Sepsis. Thus there was disease associated variation in the processing of SAA1 in ICU-Sepsis versus ICU controls or other diseases and controls.

**Conclusion:**

Specific proteins and peptides that vary between diseases might be discovered by the random and independent sampling of multiple disease and control plasma from different hospital and clinics by LC–ESI–MS/MS for storage in a relational SQL Server database and analysis with the R statistical system that will be a powerful tool for clinical research. The processing of SAA1 may play an unappreciated role in the inflammatory response to Sepsis.

## Introduction

### Analysis of septic blood by mass spectrometry

The analysis of septic blood proteins has been recently reviewed, and to date some 198 proteins have been characterized in response to septic infection [1]. Procalcitonin (CALCA) [2] C-reactive protein, interleukin (IL)-6, and soluble urokinase-type plasminogen activator receptor may serve as sepsis markers [1]. The application of 2D electrophoresis of blood fluid together with mass spectral analysis detected increased levels of serum amyloid (SAA1), inter alpha trypsin inhibitor (ITIH3) and APOJ in sepsis [3]. A sepsis model system in mice analyzed using 2D electrophoresis and MALDI-TOF/TOF MS showed changes in α-1-antitrypsin, hemopexin, kininogen, α1-acid glycoprotein, apolipoprotein A-1, and complement C3 [4]. Plasma from septic rats showed tubulin alpha 4A, vinculin, and tropomyosin to be upregulated, while complement components including C3, C6, and C9 were downregulated [5]. Pentraxin 3 (PTX3) was up regulated in LPS-treated mice and greater levels of oligomerization were associated with improved survival [6]. LPS binding protein (LPB), CRP, serum amyloid A, (SAA) and hepatocyte growth factor-like protein were up regulated in response to LPS injections [7, 8]. Plasma from systemic inflammatory response syndrome versus sepsis patients were compared by LC/LC–ESI–MS/MS that showed increased observation frequency for C4, CRP, plasminogen precursor, apolipoprotein A-II, plasma protease C1 inhibitor precursor, transthyretin precursor, while serum amyloid P-component precursor, apolipoprotein A-I precursor, antithrombin-III precursor, and serum amyloid A-4 protein precursor showed lower observation frequency [8]. The proteins NGAL and VCAM were upregulated in sepsis survivors but downregulated in non-survivors and so may be part of the host response that contributes to survival [9]. Younger adults expressed fibrinogen, and von Willebrand factor in response to sepsis while CRP, LBP, and A1ACT showed lower concentrations in older adults who developed severe sepsis but Apo M in older adults was associated with increased mortality [10]. IL-1α, IP-10, sTNF-R2, and sFAS could indicate the progression to septic shock while measurement of MMP-3, IL-1α, IP-10, sIL-2R, sFas, sTNF-R1, sRAGE, GM-CSF, IL-1β and Eotaxin separated survivors [11]. Levels of IL-6, IL-8, and MCP-1 were higher in patients that succumbed to septic shock [12]. A study of 300 septic and non-septic patients showed that the combination of age and gender with CRP and PCT levels could yield a positive diagnosis of sepsis [13]. Cell based markers such as soluble TREM1 and neutrophil CD64 expression showed some utility for sepsis diagnosis [14]. Platelets isolated from patients with severe sepsis or septic shock were compared to healthy donors and showed increased expression, including EFCAB7, actin, IL-1β, GPIX, and GPIIB [15]. Recently neutrophil associated Olfactomedin-4 (OLFM4) was indicated to be a potential marker of sepsis [16]. The apparent secretion of proteins such as Defensin alpha-1, myeloperoxidase, resistin, Orosomucoid-1, and Haptoglobin from white blood cells versus CD44 antigen, Granulins, NGAL, Serotransferrin, Catalase, and others may indicate the host response [1, 17]. Here, the selection of septic patients versus a heterogeneous population of ICU patients was employed to look for sepsis markers in the relevant background population [18, 19]. More recently, the use of two dimensional PAGE revealed that complement components, haptoglobin and ceruloplasmin amongst others show longitudinal variation over the course of septic infection [20, 21].

### Blood peptides and proteins

The endogenous peptides of human serum and plasma were first detected by highly sensitive MALDI [22–24]. The MALDI “patterns” formed by the ex vivo degradation of the major peptides of human blood fluids have been compared using complex multivariate approaches [25–29]. However, it is difficult to conceive of how multivariate analysis will function as routine diagnostic [30]. Multivariate “pattern analysis” is prone to over-interpretation of laboratory or clinical experiments and so univariate or two way ANOVA may be a more robust strategy [31–34]. Multivariate analysis provided about the same statistical power compared to the univariate ANOVA of the main feature(s) [32]. The endogenous peptides (peptidome) of human blood were first identified by MS/MS fragmentation using MALDI-Qq-TOF and LC–ESI–MS/MS with an ion trap mass spectrometer and the intensity values compared by ANOVA [32]. The endogenous peptides of blood fluid (peptidome) where no digestion enzyme was specified showed excellent agreement with the exogenously digested tryptic peptides (proteome) [35]. Random and independent sampling of the endogenous tryptic peptides from clinical plasma samples revealed individual peptides or proteins that show significant variation by standard statistical tests such as the Chi square test and ANOVA [32, 36–40]. Pre-analytical variation was exhaustively studied between fresh EDTA plasma samples on ice versus plasma samples degraded for various lengths of time to control for differences in sample handling and storage.

### Sample preparation

The sensitive analysis of blood fluids by LC–ESI–MS/MS is dependent on effective fractionation strategies, such as partition chromatography [37, 41] or organic extraction [42–45], to relieve suppression and competition for ionization, resulting in high signal to noise ratios and thus low error rates of identification and quantification [46]. Without step wise sample partition only a few high abundance proteins may be observed from blood fluid [35, 41, 42]. In contrast, with sufficient sample preparation, low abundance proteins of  ≤ 1 ng/ml could be detected and quantified in blood samples by mass spectrometry [41, 44]. Simple and single-use, i.e. disposable, preparative and analytical separation apparatus permits the identification and quantification of blood peptides and proteins with no possibility of cross contamination between patients that guarantees sampling is statistically independent [32, 35, 39, 41, 44]. Precipitation and selective extraction of the pellet [43–45, 47] was shown to be superior to precipitation and analysis of the ACN supernatant [48], ultra-filtration, [49] albumin depletion chromatography [50] or C18 partition chromatography alone [35]. Precipitating all of the polypeptides with 90% ACN followed by step-wise extraction of the peptides with mixtures of organic solvent and water was the optimal method to sensitively detect peptides from blood [42]. Here a step gradient of acetonitrile/water to extract 200 µl of EDTA plasma for analysis by LC–ESI–MS/MS showed a high signal to noise ratio [42] and resulted in the confident identification of tryptic peptides [39] from ICU-Sepsis versus ICU Control samples.

### Computation and statistics

Partition of each clinical sample into multiple sub-fractions, that each must be randomly and independently sampled by analytical C18 LC–ESI–MS/MS, provides sensitivity [42] but also creates a large computational challenge. Previously the 32-bit computer power was lacking to identify and compare all the peptides of all the proteins of the many sub-factions from each patient in a large multisite clinical experiment [51]. The MS/MS spectra from random and independent sampling of peptides from thousands of LC–ESI–MS/MS experiments from multiple clinical treatments and sites may be fit to peptides by the X!TANDEM and SEQUEST algorithms [52, 53]. The observation frequency and precursor intensity compared across treatments using a 64 bit server SQL SERVER shows excellent data compression and relation for classical analysis with the R statistical system [36, 39]. The presence of powerful, multi-core 64-bit computation now permits the large scale random and independent sampling of blood peptides and proteins by LC–ESI–MS/MS with followed by peptide correlation and classical analysis in SQL SERVER with the R statistical system. The protein p-values and FDR q-values were computed from organic extraction or chromatography of blood fluid and the peptide-to-protein distribution of the precursor ions of greater than ~ 10,000 (E4) counts were compared to a null (i.e. known false positive) model of noise or computer generated random MS/MS spectra [31, 37, 39, 54–56]. Peptides may be identified from the fit of MS/MS spectra to peptide sequences by X!TANDEM [52] that permits the accurate estimate of the type I error rate (*p-*value) of protein identification that may be corrected by the method Benjamini and Hochberg [57] to yield the FDR (q-value) [39, 42, 54]. Simulations using random or noise MS/MS spectra distributions may be used to control the type I error of experimental MS/MS spectra correlations to tryptic peptides: The peptide and protein observation counts (frequency) may be analyzed using classical statistic methods such as Chi square analysis [55, 58]. A log_10_ transformation of precursor intensity yields a normal distributions that permits comparison of peptide and proteins expression levels by ANOVA [37, 38]. The SQL Server system permits the direct interrogation of the related data by the open source R statistical system without proteomic-specific software packages. The use of SQL/R has permitted the detailed statistical analysis of randomly and independently sampled LC–ESI–MS/MS data from multiple hospitals in parallel that would be requisite for a multisite clinical trial [58, 59].

### ICU-Sepsis versus ICU Control and other disease and controls

Here, the combination of step wise organic partition [42], random and independent sampling by nano electrospray LC–ESI–MS/MS [39], and 64 bit computation with SQL SERVER/R [36] permitted the sensitive detection of peptides and/or phosphopeptides. Thus, variation in the cleavage of parent protein chains and complexes, from human plasma were compared between ICU-Sepsis versus ICU Control alongside other disease and normal plasma by the classical statistical approaches of the Chi square test of observation frequency, STRING analysis of the identified proteins and univariate or two-way ANOVA of protein and peptide intensity [31, 32, 37, 38].

## Materials and methods

### Materials

Anonymous human EDTA plasma with no identifying information were received and analyzed under the Ryerson Ethical Review Board Protocol REB 2015-207: ICU-Sepsis versus ICU Control EDTA plasma were obtained from Clinical Evaluation Research Unit, Kingston General Hospital, Kingston Ontario Canada; Ovarian and breast cancer samples were obtained from the Ontario Tumor bank of the Ontario Institute of Cancer Research, Toronto Ontario; Heart attack (venous and arterial) versus pre-operative orthopedic surgery controls were obtained from St Joseph’s Hospital of McMaster University; Multiple sclerosis Alzheimer’s dementia and institution-matched normals were obtained from Amsterdam University Medical Centers, Vrije Universiteit Amsterdam; In addition, EDTA plasma samples collected onto ice as a baseline degradation controls were obtained from IBBL Luxembourg [39, 40]. C18 zip tips were obtained from Millipore (Bedford, MA), C18 HPLC resin was from Agilent (Zorbax 300 SB-C18 5-micron). Solvents were obtained from Caledon Laboratories (Georgetown, Ontario, Canada). All other salts and reagents were obtained from Sigma-Aldrich-Fluka (St Louis, MO) except where indicated. The level of replication in the LC–ESI–MS–MS experiments was typically between 9 to 26 independent patient plasma samples (Additional file 1: Table S1).

### Sample preparation

A total of 10 ICU-Sepsis versus 9 ICU Control Human EDTA plasma samples (200 μl) were precipitated with 9 volumes of acetonitrile (90% ACN) [44], followed by extraction of the pellet using a step gradient to achieve selectivity across sub-fractions and thus greater sensitivity [42]. Disposable plastic 2 ml sample tubes and plastic pipette tips were used to handle samples. The acetonitrile suspension was separated with a centrifuge at 12,000 RCF for 5 min. The acetonitrile supernatant, that contains few peptides, was collected, transferred to a fresh sample tube and dried in a rotary lyophilizer. The organic precipitate (pellet) that contains a large total amount of endogenous polypeptides [44] was manually re-suspended using a step gradient of increasing water content to yield 10 fractions from those soluble in 90% ACN to 10% ACN, followed by 100% H_2_O and then 5% formic acid [42]. The extracts were clarified with a centrifuge at 12,000 RCF for 5 min. The extracted sample fractions were dried under vacuum in a rotary lyophilizer and stored at − 80 °C for subsequent analysis.

### Preparative C18 chromatography

Preparative C18 separation provided the best results for peptides and phosphopeptides analysis in a “blind” test [60]. Solid phase extraction with C18 for LC–ESI–MS/MS was performed as previously described [32, 35, 41, 44, 47]. The C18 chromatography resin (Zip Tip) was wet with 65% acetonitrile before equilibration in water with 5% formic acid. The plasma extract was dissolved in 200 μl of 5% formic acid in water. The resin was washed with at least five volumes of the same binding buffer. The resin was eluted with ≥ 3 column volumes of 65% acetonitrile (2 µl) in 5% formic acid. In order to avoid cross-contamination the preparative C18 resin was discarded after a single use.

### Lc–ESI–MS/MS

In order to entirely prevent any possibility of cross contamination, a new disposable nano analytical HPLC column and nano emitter was fabricated for recording each patient sample-fraction set. The ion traps were cleaned and tested for sensitivity with angiotensin and glu fibrinogen prior to recordings. The new column was conditioned and quality controlled with a mixture of three non-human protein standards [31] using a digest of Bovine Cytochrome C, Yeast alcohol dehydrogenase (ADH) and Glycogen Phosphorylase B to confirm the sensitivity and mass accuracy of the system prior to each patient sample set. The statistical validity of the linear quadrupole ion trap for LC–ESI–MS/MS of human plasma [42] was in agreement with the results from the 3D Paul ion trap [31, 37, 55, 56]. The stepwise extractions were collected and desalted over C18 preparative micro columns, eluted in 2 µl of 65% ACN and 5% formic acid, diluted ten-fold with 5% formic acid in water and 5% ACN, and immediately loaded manually into a 20 μl metal sample loop before injecting onto the analytical column via a Rheodyne injector. Endogenous peptide samples were analyzed over a discontinuous gradient generated at a flow rate of ~ 10 micro litres per minute with an Agilent 1100 series capillary pump and split upstream of the injector during recording to about ~ 200 nl per minute. The separation was performed with a C18 (150 mm × 0.15 mm) fritted capillary column. The acetonitrile profile was started at 5%, ramped to 12% after 5 min and then increased to 65% over ~ 90 min, remained at 65% for 5 min, decreased to 50% for 15 min and then declined to a final proportion of 5% prior to injection of the next step fraction from the same patient. The nano HPLC effluent was analyzed by ESI ionization with detection by MS and fragmentation by MS/MS with a linear quadrupole ion trap [61]. The instrument was set to collect the precursors for up to 200 milli seconds prior to MS/MS fragmentation with up to four independent MS/MS fragmentations per precursor ion. Individual, independent samples from disease, normal and ice cold control were precipitated, fractionated over a step gradient and collected over C18 for manual injection.

### Correlation analysis

Correlation analysis of ion trap data was performed using a goodness of fit test by X!TANDEM [52] and by cross-correlation using SEQUEST [62] on separate servers to match tandem mass spectra to peptide sequences from the Homo sapiens RefSeq, Ensembl, SwissProt, including hypothetical proteins XP or Genomic loci [35, 36, 63]. Endogenous peptides with precursors greater than 10,000 (E4) arbitrary counts were searched only as fully tryptic peptides and/or phosphopeptides, the results were combined, and compared in SQL Server/R. The X!TANDEM default ion trap data settings of ± 3 m/z from precursor peptides considered from 300 to 2000 m/z with a tolerance of 0.5 Da error in the fragments were used [37, 41, 52, 55, 56, 64]. The best fit peptide of the MS/MS spectra to fully tryptic and/or phospho-tryptic peptides at charge states of + 2 versus + 3 were accepted with additional acetylation, or oxidation of methionine and with possible loss of water or ammonia. The resulting accession numbers, actual and estimated masses, correlated peptide sequences, peptide intensity and protein scores, resulting protein sequences and other associated data were captured and assembled together in an SQL Server relational database [36].

### Data sampling, sorting, transformation and visualization

The linear quadrupole ion trap provided the precursor ion intensity and m/z values plus the peptide fragment MS/MS spectra. The peptides and proteins were identified from MS/MS spectra by X!TANDEM and were counted by the SEQUEST algorithm. The large number of redundant correlations to each MS/MS at different charge states or to different sequences may be a source of type I error that may be filtered out by a complex key in SQL Server. The MS and MS/MS spectra together with the results of the X!TANDEM and SEQUEST algorithms were parsed into an SQL Server database and filtered [36] before statistical and graphical analysis with the generic R data system [31, 36–38, 63]. The peptide to protein correlation frequency counts for each gene symbol were summed over sepsis versus the matched control to correct the observation frequency for the Chi square test using Eq. 1:

1 $${{\left( {{\text{ICU}}\_{\text{Sepsis}} - {\text{ICU}}\_{\text{Control}}} \right)^{2} } \mathord{\left/ {\vphantom {{\left( {{\text{ICU}}\_{\text{Sepsis}} - {\text{ICU}}\_{\text{Control}}} \right)^{2} } {\left( {{\text{ICU}}\_{\text{Control}} + 1} \right)}}} \right. \kern-0pt} {\left( {{\text{ICU}}\_{\text{Control}} + 1} \right)}}$$

The precursor intensity data for MS/MS spectra were log_10_ transformed, tested for normality and analyzed across institution/study and diseases verses controls by means, standard errors and ANOVA [31, 37, 38]. The entirely independent analysis of the precursor intensity using the rigorous ANOVA with Tukey–Kramer HSD test versus multiple controls was achieved using a 64-bit R server.

## Results

Partition of plasma samples using differential solubility in organic/water mixtures combined with random and independent sampling by LC–ESI–MS/MS detected peptides and proteins that were more frequently observed and/or showed greater intensity in ICU-Sepsis versus ICU Control. Here four independent lines of evidence, Chi square analysis of observation frequency, previously established structural/functional relationships from STRING, ANOVA analysis of peptide intensity, and agreement with the previous genetic or biochemical experiments, all indicated that there was significant variation in the peptides of ICU-Sepsis patients compared to ICU Control and other diseases or normal plasma samples.

### LC–ESI–MS/MS

The pool of endogenous tryptic (TRYP) and/or tryptic phosphopeptides (TRYP STYP) were randomly and independently sampled without replacement by liquid chromatography, nano electrospray ionization and tandem mass spectrometry (LC–ESI–MS/MS) [39] from ICU-Sepsis vs ICU Control, other disease and normal plasma, and ice cold controls to serve as a baseline [40, 65]. Some 15,968,550 MS/MS spectra ≥ E4 intensity counts were correlated by the SEQUEST and X!TANDEM algorithms that resulted in a total of 19,197,152 redundant matches of MS/MS spectra to peptides in proteins. The redundant correlations from SEQUEST were filtered to retain only the best fit by charge state and peptide sequence in SQL Server to avoid re-use of the same MS/MS spectra. The filtered results were then analyzed by the generic R statistical system in a matrix of disease and controls that reveals the set of blood peptides and proteins specific to each disease state. The statistical validity of the extraction and sampling system were previously established by computation of protein gene symbols p-values and FDR corrected q-values by the method of Benjamini and Hochberg [57] and frequency comparison to false positive noise or random spectra [39, 42].

### Frequency correction

**Fig. 1:**
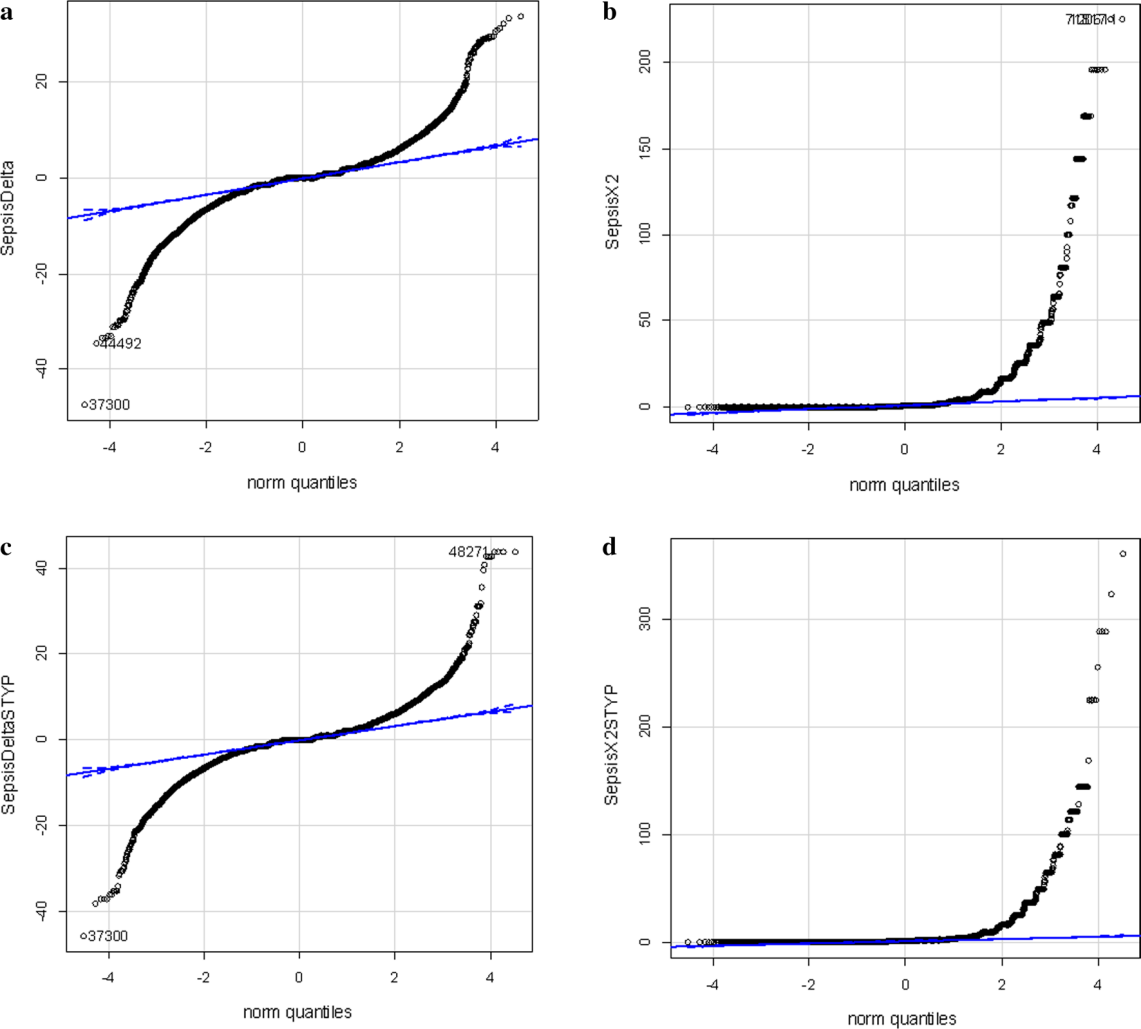
Quantile plots of the corrected difference in observation frequency and Chi square values of the ICU-Sepsis (n ≥ 10) versus ICU Control (n ≥ 9). **a** Quantile plot of the observation frequency of tryptic peptides from ICU-Sepsis versus ICU Control. **b** χ^2^ plot of the observation frequency of tryptic peptides from ICU-Sepsis versus ICU Control cancer tryptic peptides. **c** Quantile plot of the observation frequency of tryptic STYP peptides from ICU-Sepsis versus ICU Control. **d** χ^2^ plot of the observation frequency of tryptic STYP peptides from ICU-Sepsis versus ICU Control tryptic peptides

Chi square (χ^2^) may be used to compare discrete, “counting” variables such as observation frequency. A total of 486,367 MS/MS ≥ E4 counts were collected from ICU-Sepsis samples and 424,591 MS/MS ≥ E4 counts were collected from ICU Control plasma and these sums were used to correct observation frequency. Similar results were obtained from comparison corrected on the basis of total correlations (not shown). Many proteins show large increases or decreases in observation frequency between ICU Sepsis versus the matched ICU Control resulting in large Chi square values (Fig. 1).

### Comparison of sepsis to matched control by Chi square analysis

A set of ~ 50 gene symbols showed a substantial difference ≥ 9 counts and a Chi square (χ^2^) values of ≥ 9 between ICU-Sepsis versus the matched ICU Control. For χ^2^ analysis the tryptic peptides (TRYP) were computed independently from the phospho-tryptic peptides. Specific peptides and/or phosphopeptides from cellular proteins, membrane proteins, nucleic acid binding proteins, signaling factors, metabolic enzymes and others including uncharacterized proteins showed significantly greater observation frequency in ICU-Sepsis. In agreement with the literature, peptides from ITIH3, SAA2, SAA1, and FN1 showed variation in observation frequency between ICU-Sepsis versus ICU Control. The Chi square analysis showed some cellular proteins with large changes in frequency by Chi square (χ^2^ > 9, p ≤ 0.00389) from tryptic (TYRP) peptides such as POTEB, CTNNA1, U2SURP, KIF24, NLGN2, KSR1, GTF2H1, KIT, RPS6KL1, VAV2, HSPA7, SMC2, TCEB3B, ZNF300, SUPV3L1, ADAMTS20, LAMB4, MCCC1, SUPT6H, SCN9A, SBNO1, EPHA1, ABLIM2, cB5E3.2, EPHA10, GRIN2B, HIVEP2, CCL16, TKT, LRP2 and TMF1 amongst others. Similarly, increased tryptic phosphopeptides were observed from POM121C, SCN8A, TMED8, NSUN7, SLX4, MADD, DNLZ, PDE3B, UTY, DEPDC7, MTX1, MYO1E, RXRB, SYDE1, FN1, PUS7L, FYCO1, USP26, ACAP2, AHI1, KSR2, LMAN1, ZNF280D and SLC8A2 amongst others (Table 1). Observation frequency may be the best measure of relative abundance [66] and the full list of Chi square results are found in Additional files 1, 2, 3 that is the most important result of this study.

**Table 1:** Sepsis specific proteins detected by fully tryptic peptides and/or fully tryptic phosphopeptides that show a Chi square (χ^2^) value of ≥ 45

TRYP_gene_symbol	Sepsis_X2	Gene_symbol_n	STYP_gene_symbol	STYP_X2	Gene_symbol_n
POTEB	210.5	2	POM121C	324.0	1
POTEB2	196.0	1	SCN8A	273.0	4
CTNNA1	178.7	3	TMED8	156.5	2
U2SURP	178.0	3	NSUN7	132.5	2
KIF24	146.9	4	SLX4	127.9	1
NLGN2	144.0	2	MADD	121.1	26
ITIH3	139.4	5	DNLZ	121.0	1
KSR1	132.9	8	PDE3B	107.0	3
GTF2H1	121.0	2	UTY	105.6	57
KIT	121.0	1	SAA2	103.1	1
RPS6KL1	114.5	7	DEPDC7	100.0	2
VAV2	111.8	4	MTX1	100.0	1
HSPA7	100.0	1	MYO1E	100.0	1
SMC2	100.0	1	RXRB	90.5	6
TCEB3B	100.0	1	SYDE1	84.2	4
ZNF300	100.0	1	SAA1	71.0	4
SUPV3L1	83.8	2	FN1	67.5	28
ADAMTS20	76.6	2	PUS7L	64.2	3
LAMB4	76.6	2	FYCO1	63.6	3
SAA2	71.5	1	USP26	59.8	2
SAA1	71.1	4	ACAP2	55.5	3
MCCC1	69.1	2	AHI1	55.5	1
SUPT6H	65.3	2	DKFZp686O12165	55.5	1
SCN9A	61.5	4	KSR2	55.5	3
SBNO1	60.3	2	LMAN1	55.5	1
EPHA1	57.5	3	ZNF280D	55.2	9
ABLIM2	55.7	2	SLC8A2	50.9	2
cB5E3.2	55.7	1	ITPR2	49.8	2
EPHA10	55.7	2	GPHN	49.8	8
GRIN2B	55.7	1	HES6	48.6	1
HIVEP2	54.4	1	OSBPL7	48.6	3
CCL16	53.8	1	TRPM6	47.2	8
TKT	52.4	4	MSR1	46.7	5
LRP2	52.3	2	C17orf25	46.3	1
TMF1	51.9	2	GPHRYN	46.3	1

The full set of gene symbols from tryptic and phospho tryptic peptides and the resulting STRING analysis may be found in Additional files 1, 2, 3: Tables S1, S2 and S3

### STRING network analysis

**Fig. 2:**
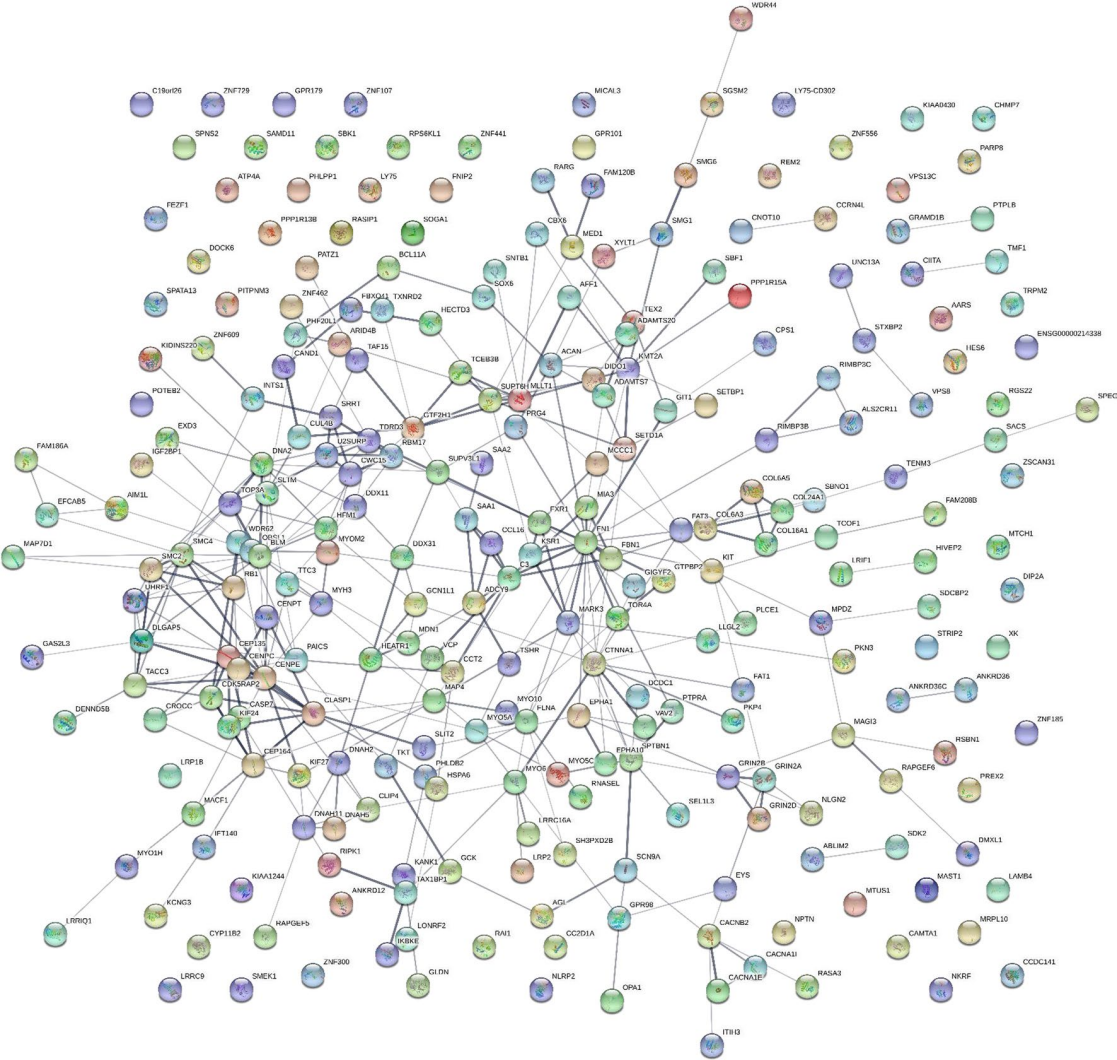
The sepsis STRING network where tryptic (TRYP) peptide frequency difference greater than 10 and Chi square χ^2^ ≥ 9 (p < 0.01). See Additional files 1, 3: Tables S1 and S3

**Fig. 3:**
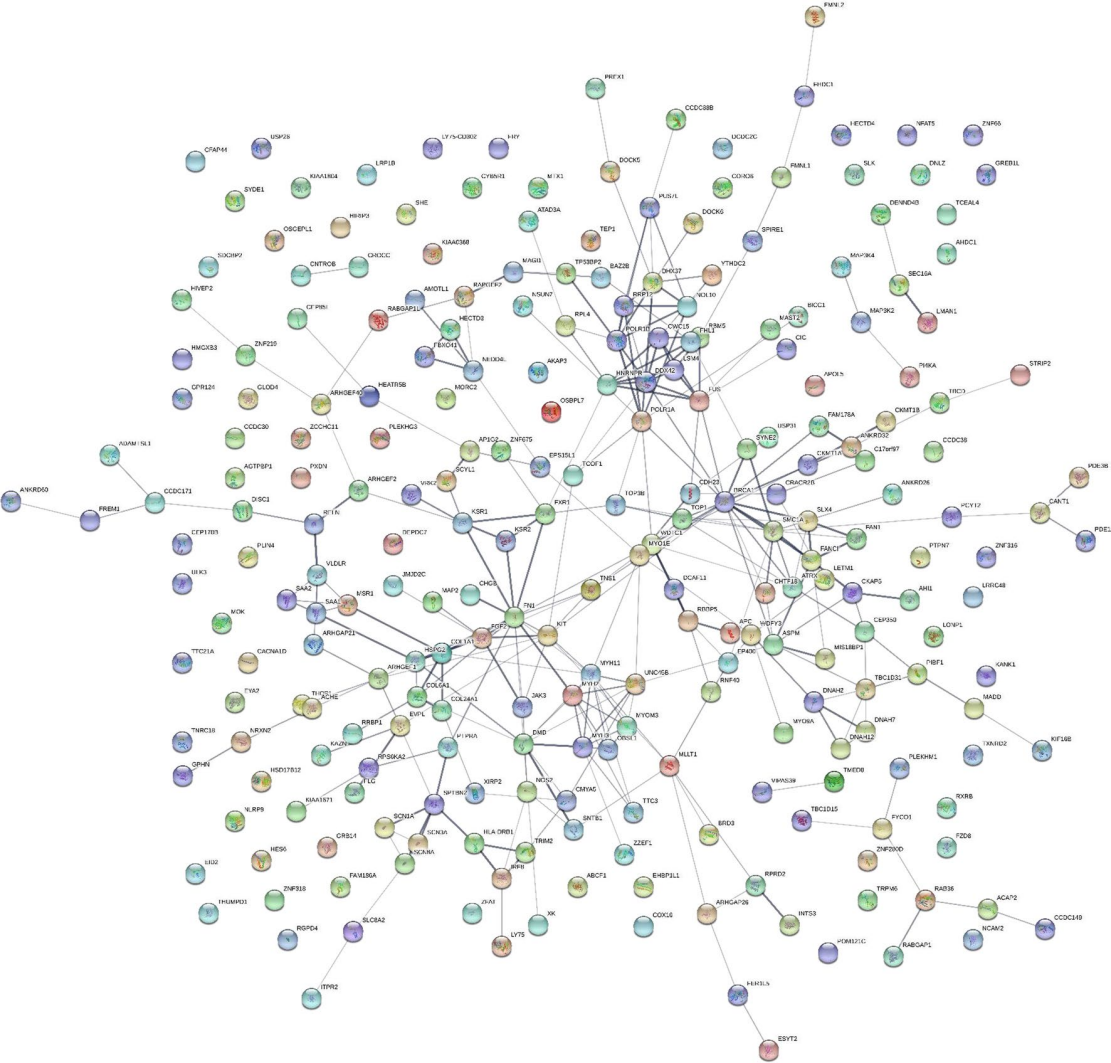
The sepsis STRING network where tryptic and/or phospho-tryptic (STYP) peptide frequency difference greater than 10 and Chi square χ^2^ ≥ 9 (p < 0.01). See Additional files 2, 3: Table S2, S3

Taking the Gene symbols that varied between ICU-Sepsis versus ICU Control by Chi square (χ^2^) revealed a network of proteins that show a dense and complex set of interconnections with 484 nodes connected by 1147 edges with a PPI enrichment p-value of < 1.0e−16 (see Additional file 1: Table S1). The analysis of the proteins with high observation frequencies showed proteins associated with phagocytic functions such as actin-mysoin and tubulin- dynein cytoskeleton and supramolecular fiber networks, motility, contractile proteins and proteins associated with fila podia or cell projections, the release of membrane bound organelle, lumen contents, and ATP/GTP binding and hydrolysis proteins involved in metabolic energy or cellular regulation (see Additional files 1, 2, 3). For the purposes of illustration the proteins that showed at least 9 greater observations (Delta) and χ^2^ values greater than 9 (p ≤ 0.003) are shown separate as tryptic (TRYP) versus tryptic or phospho-tryptic peptides (STYP) (Fig. 2 and 3). The full list of Gene Symbols from tryptic peptides, phospho tryptic peptides and the resulting STRING analysis may be found in Additional files 1, 2, 3: Table S1, S2 and S3.

### Quantile box plots and ANOVA analysis across disease and control treatments

**Fig. 4:**
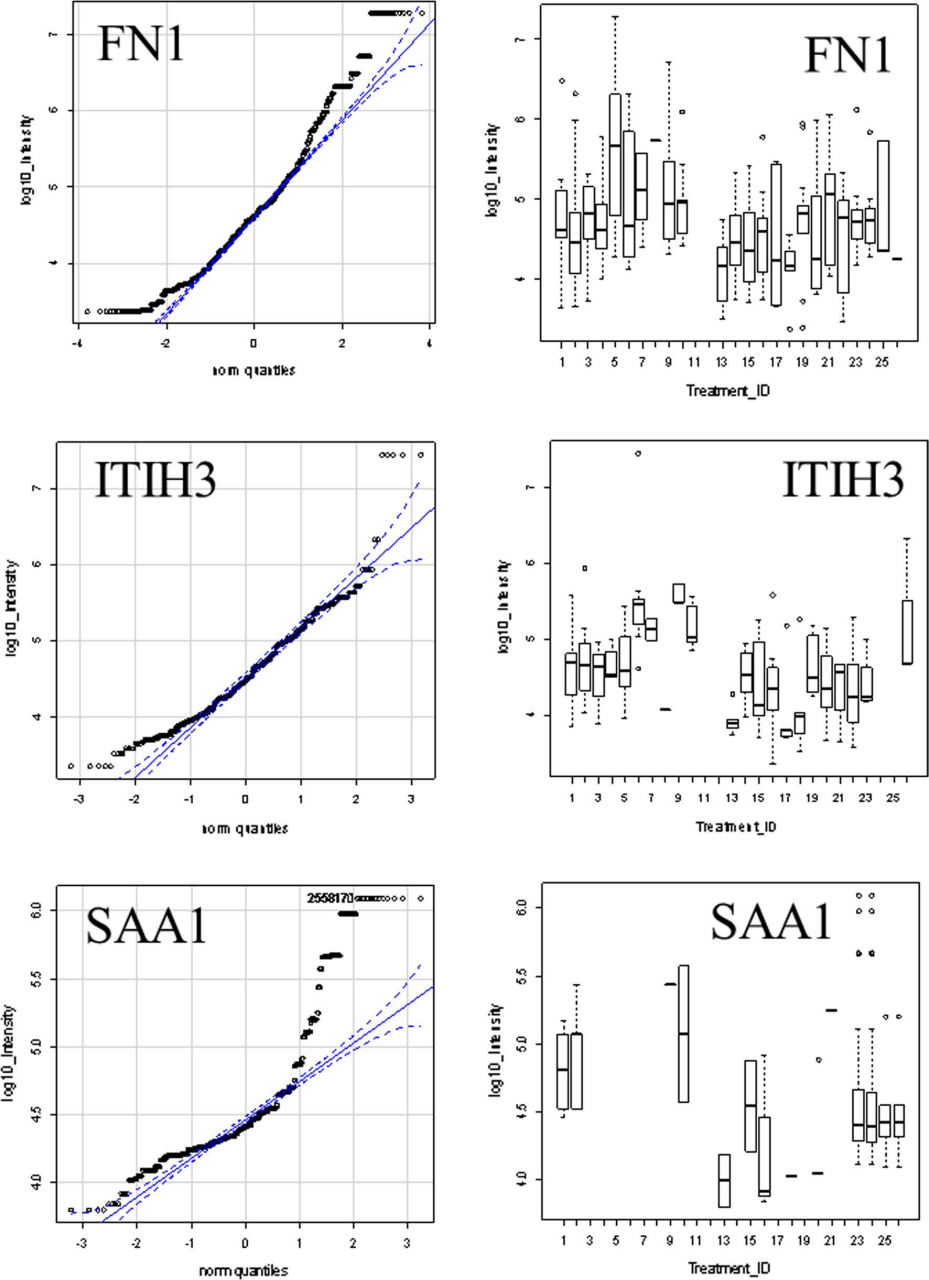
The distributions of log_10_ precursor intensity by quantile and box plots of serum proteins APOE, ITIH4, and C3 across the disease and control treatments. Treatment ID numbers: 1, Alzheimer normal; 2, Alzheimer’s normal control STYP; 3, Alzheimer’s dementia; 4, Alzheimer’s dementia STYP; 5, Cancer breast; 6, Cancer breast STYP; 7, Cancer control; 8, Cancer control STYP; 9, Cancer ovarian; 10, Cancer ovarian STYP; 11, Ice Cold; 12, Ice Cold STYP; 13, Heart attack Arterial; 14 Heart attack Arterial STYP; 15, Heart attack normal control, 16, Heart attack normal Control STYP; 17, Heart attack; 18, Heart attack STYP; 19, Multiple Sclerosis normal control; 20, Multiple sclerosis normal control STYP; 21, Multiple sclerosis; 22, Multiple Sclerosis STYP, 23, Sepsis; 24, Sepsis STYP; 25, Sepsis normal control; 26, Sepsis normal control STYP. There was significant effects of treatments and peptides by two-way ANOVA. Analysis of the proteins shown across treatments produced a significant F Statistic by one-way ANOVA

**Fig. 5:**
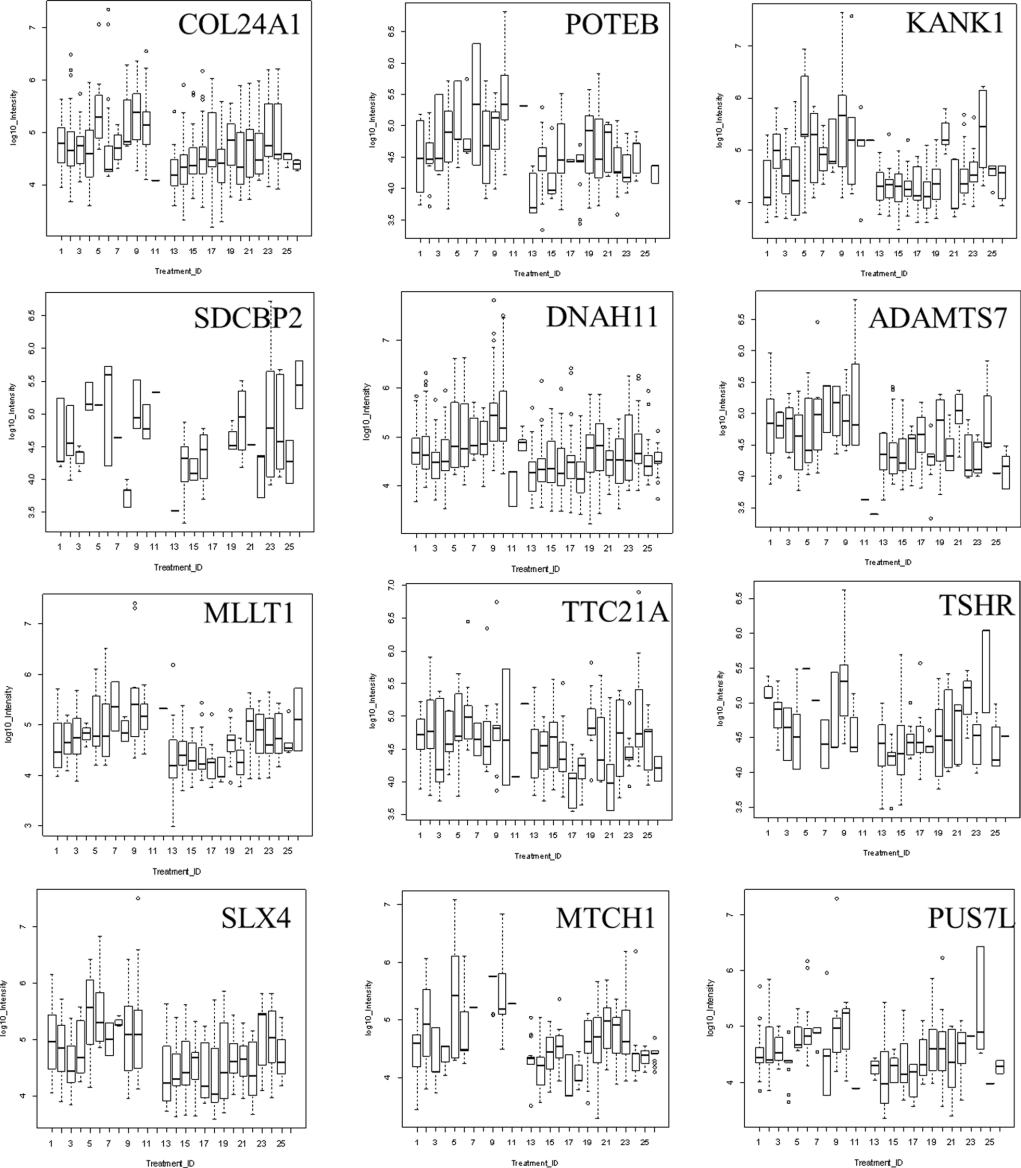
The distributions of log_10_ precursor intensity by box plots of the cellular proteins COL24A1, POTEB, KANK1, SDCBP2, DNAH11, ADAMTS7, MLLT1, TTC21A, TSHR, SLX4, MTCH1, and PUS7L across the disease and control treatments. Treatment ID numbers: 1, Alzheimer normal; 2, Alzheimer’s normal control STYP; 3, Alzheimer’s dementia; 4, Alzheimer’s dementia STYP; 5, Cancer breast; 6, Cancer breast STYP; 7, Cancer control; 8, Cancer control STYP; 9, Cancer ovarian; 10, Cancer ovarian STYP; 11, Ice Cold; 12, Ice Cold STYP; 13, Heart attack Arterial; 14, Heart attack Arterial STYP; 15, Heart attack normal control, 16, Heart attack normal Control STYP; 17, Heart attack; 18, Heart attack STYP; 19, Multiple Sclerosis normal control; 20, Multiple sclerosis normal control STYP; 21, Multiple Sclerosis; 22, Multiple sclerosis STYP, 23, Sepsis; 24, Sepsis STYP; 25, Sepsis normal control; 26, Sepsis normal control STYP. There was significant effects of treatments and peptides by two-way ANOVA. Analysis of the proteins shown across treatments produced a significant F Statistic by one-way ANOVA

ANOVA may be an independent method to confirm the potential utility of peptides from Gene symbols that showed increased observation frequency by Chi square. Some proteins that showed greater observation frequency in sepsis also showed significant variation in protein or peptide precursor intensity compared to the sepsis controls and/or other disease and normal EDTA plasma by quantile box plots and/or ANOVA comparison. The mean precursor intensity values from gene symbols that varied by Chi square (χ^2^ > 9) from tryptic and/or phospho tryptic were subsequently analyzed by univariate ANOVA in R to look for proteins that showed significant variation in precursor intensity values across treatments [32, 38] (Figs. 4 and 5). Common plasma proteins including ITIH3, FN1 and SAA1 showed significant variation in average peptide log_10_ intensity across treatments (Fig. 4). Analysis of the precursor intensity by quantile box plots and ANOVA confirmed significant variation in cellular proteins with increased observation frequency in sepsis such as COL24A1, POTEB, KANK1, SDCBP2, DNAH11, ADAMTS7, MLLT1, TTC21A, TSHR, SLX4, MTCH1, and PUS7L (Fig. 5).

### Processing of SAA1 in sepsis versus matched controls

**Fig. 6:**
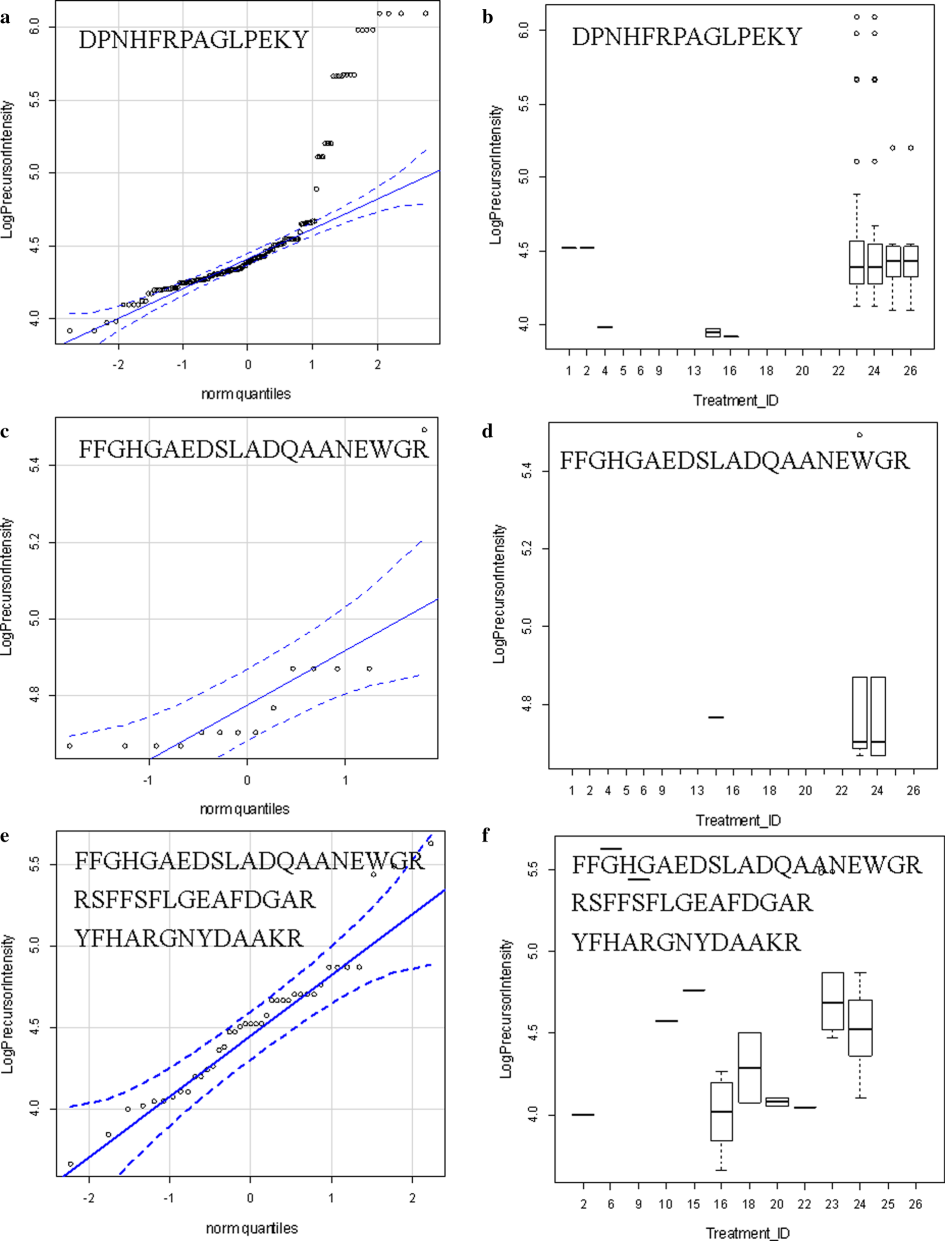
SAA1 peptides including the COOH terminal PNHFRPAGLPEKY peptide versus all other more NH2 terminal peptides. **a** D/PNHFRPAGLPEKY quantile plot. **b** D/PNHFRPAGLPEKY intensity box plot. **c** SAA1 amino terminal peptide FFGHGAEDSLADQAANEWG quantile plot. **d** SAA1 amino terminal peptide FFGHGAEDSLADQAANEWG box plot. **e** SAA1 amino terminal grouped peptide FFGHGAEDSLADQAANEWG, RSFFSFLGEAFDGAR & YFHARGNYDAAKR quantile plot. **f** SAA1 amino terminal grouped peptide FFGHGAEDSLADQAANEWG, RSFFSFLGEAFDGAR and YFHARGNYDAAKR box plot. Treatment ID numbers: 1, Alzheimer normal; 2, Alzheimer’s normal control STYP; 3, Alzheimer’s dementia; 4, Alzheimer’s dementia STYP; 5, Cancer breast; 6, Cancer breast STYP; 7, Cancer control; 8, Cancer control STYP; 9, Cancer ovarian; 10, Cancer ovarian STYP; 11, Ice Cold; 12, Ice Cold STYP; 13, Heart attack Arterial; 14, Heart attack Arterial STYP; 15, Heart attack normal control, 16, Heart attack normal Control STYP; 17, Heart attack; 18, Heart attack STYP; 19, Multiple Sclerosis normal control; 20, Multiple Sclerosis normal control STYP; 21, Multiple sclerosis; 22, Multiple sclerosis STYP, 23, Sepsis; 24, Sepsis STYP; 25, Sepsis normal control; 26, Sepsis normal control STYP. There was significant effects of treatments and peptides by two-way ANOVA

**Fig. 7:**
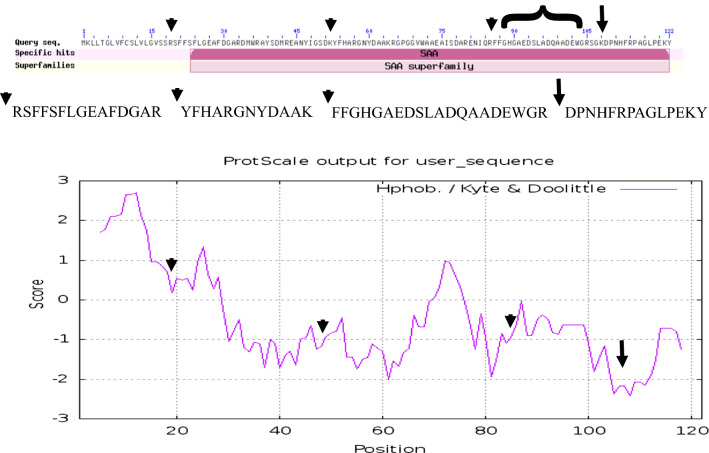
The primary structure and hydrophilicity plot of SAA1 protein accession AAH07022.1. The long arrow shows the cleavage site of the fully tryptic peptides sequence DPNHFRPAGLPEKY on the COOH terminus adjacent to the cystatin C binding site from AA 86-104 (bracket) that resolved ICU and Sepsis-ICU from all other treatments and controls. The short arrows show the location of cleavages NH2 terminal of the cystatin C binding site

The SAA1 protein that best fit the MS/MS spectra observed from human plasma was accession AAH07022.1. The observation frequency of peptides from SAA1 was much higher in ICU-Sepsis compared to any other disease of control treatment (Table 2). The processing of SAA1 including the cleavage of the C-terminal tryptic peptide DPNHFRPAGLPEKY from the most hydrophilic point of SAA1 on the COOH side of the Cystatin C binding was most apparent in ICU-Sepsis patients compared to all other diseases and controls. Additional cleavages of SAA1 on the NH2 terminus side of the Cystatin Binding Site were observed in ICU-Sepsis. An exopeptidase activity apparently removed the N-terminal aspartic acid, D, from some of the released peptides that we represent as D/PNHFRPAGLPEKY. Thus there was disease associated variation in the processing of SAA1 in ICU-Sepsis versus ICU controls or other diseases and normal (Table 2). The cleavage of the terminal peptide D/PNHFRPAGLPEKY from the most hydrophilic point of SAA1 on the COOH side of the cystatin binding site (Figs. 6 and 7) was most frequently observed in ICU-Sepsis compared to all other treatments. Septic patients showed the cleavage of _109_D/PNHFRPAGLPEKY_122_ from the COOH terminus plus additional cleavages on the NH2 side of the Cystatin C binding site of SAA1 at the tryptic cleavage site _20_SFFSFLGEAFDGAR_33_, _53_YFHARGNYDAAK_64_ and the peptide _86_FFGHGAEDSLADQAADEWGR_105_ that overlaps the cystatin binding site (Figs. 6 and 7). Thus, the processing of SAA1 in ICU-Sepsis patients apparently varied compared to all other diseases and controls.

**Table 2:** The analysis of redundant mean peptide intensity and observation frequency of the full length and truncated SAA1 protein

Treatment_ID	Full length SAA1			Truncated SAA1		
	Peptide_intensity	se (mean)	Data: n	Peptide_intensity	se (mean)	Data: n
1	104,227.4	32,319.6	7	104,227.4	32,319.6	7
2	89,372.6	50,226.2	5	89,372.6	50,226.2	5
4	20,655.1	11,064.5	2	20,655.1	11,064.5	2
5	2,918,290.3	NA	1	2,918,290.3	NA	1
6	426,255.1	NA	1	426,255.1	NA	1
9	174,935.2	100,095.9	2	174,935.2	100,095.9	2
10	335,900.6	209,560.4	4	335,900.6	209,560.4	4
13	19,326.3	11,375.7	4	19,326.3	11,375.7	4
15	31,441.6	8916.8	9	31,441.6	8916.8	9
16	18,908.6	7187.1	10	18,908.6	7187.1	10
17	20,049.3	8467.0	3	20,049.3	8467.0	3
18	138,654.0	120,611.1	4	138,654.0	120,611.1	4
19	34,821.0	NA	1	34,821.0	NA	1
20	140,968.2	112,007.7	5	140,968.2	112,007.7	5
21	178,542.8	NA	1	178,542.8	NA	1
22	11,157.6	NA	1	11,157.6	NA	1
23	109,959.2	28,031.2	80	55,875.9	13,232.7	21
24	106,006.4	28,315.5	79	37,558.3	3945.8	20
25	43,369.7	9799.9	19	46,682.4	8607.8	4
26	42,592.9	10,314.1	18	43,126.0	10,418.8	3

Treatment ID numbers: 1, Alzheimer normal; 2, Alzheimer’s normal control STYP; 3, Alzheimer’s dementia; 4, Alzheimer’s dementia STYP; 5, Cancer breast; 6, Cancer breast STYP; 7, Cancer control; 8, Cancer control STYP; 9, Cancer ovarian; 10, Cancer ovarian STYP; 11, Ice Cold; 12, Ice Cold STYP; 13, Heart attack Arterial; 14, Heart attack Arterial STYP; 15, Heart attack normal control, 16, Heart attack normal Control STYP; 17, Heart attack; 18, Heart attack STYP; 19, Multiple Sclerosis normal control; 20, Multiple sclerosis normal control STYP; 21, Multiple sclerosis; 22, Multiple Sclerosis STYP, 23, Sepsis; 24, Sepsis STYP; 25, Sepsis normal control; 26, Sepsis normal control STYP

## Discussion

A simple and direct strategy to discover variation in peptides or proteins specific to ICU-Sepsis may be to compare them to ICU control, alongside other disease and normals under identical conditions. The aim and objective of this study was proof of concept towards a method to compare the endogenous tryptic peptides of ICU-Sepsis to those from ICU Control and other disease or normals by random and independent sampling with a set of robust and sensitive linear quadrupole ion traps where the results were collected in an SQL Server for analysis with the R statistical system. Random and independent sampling of peptides from step-wise fractionation followed by LC–ESI–MS/MS is a time and manual labor intensive approach that is sensitive, direct, and rests on few assumptions [39, 67]. High signal to noise ratio of blood peptides is dependent on sample preparation to break the sample into many sub-fractions to relieve competition and suppression of ionization and thus achieve sensitivity [35, 41, 42] but then requires large computing power to re-assemble the sub-fractions, back into individual patient samples within treatments [36, 42, 67]. The approach shows great sensitivity and flexibility but relies on the fit of MS/MS spectra by X!TANDEM and SEQUEST [52, 53] to assign peptide identity and statistical analysis of precursor ion counts and intensity by Chi square and ANOVA and so is computationally intensive [31, 36]. The careful study of variation over time, and under various storage and preservation conditions, seems to rule out pre-clinical variation as the most important source of variation between ICU-Sepsis versus ICU Control or other disease and control treatments [39, 40, 65].

### Discovery by Chi square (χ^2^) analysis of observation frequency

The SQL Server and R statistical system permits the rapid statistical and graphical analysis of the data at the level of Gene symbols, proteins or peptides. The Chi square (χ^2^) algorithm is simple means to compute the differences in observation frequency that can be extensively applied to all gene symbols to reveal peptides and proteins that apparently differ between two treatments. Examining the observation frequency by Chi square between ICU-Sepsis versus ICU Control revealed a large set of proteins that not been previously discovered with respect to sepsis in the literature. Analysis of the protein and peptides frequently observed in sepsis across all twelve disease and controls clinical sample sets using box plots and ANOVA was a direct means to look for factors specific to sepsis that might play a role in the host response to septic infection that have not been previously considered in the literature.

### STRING analysis

The large number of edge connections between the gene symbols with increased observation frequency in sepsis indicates the proteins observed show biological and protein- interactions and were not a random assemblage of factors consistent with bone fide biological variation between the ICU-Sepsis versus ICU Control treatments. The network analysis showed proteins associated with cell motility and contraction, formation of cellular extensions, secretion of the lumen contents of membrane bound organelles expected in inflammation and the host response to bacteria that indicated a role for the innate immune response of leukocytes [9, 12, 20, 21] and was consistent with regulation of leukocytes by cytokines, chemokines and other potent regulatory factors (see Additional file 1: Table S1) [10].

### ICU-Sepsis versus ICU Control alongside other diseases and control by ANOVA

Proteins that showed increased observation frequency in ICU-Sepsis versus ICU Control by Chi square (χ^2^) were then analyzed across all disease and controls treatments by box plots, quantile plots and ANOVA. The analysis of mean precursor intensity [32, 37, 38] is complicated by the different peptide sequences within each protein that required two way ANOVA [31] and peptides shared by different proteins, or tryptic versus phospho-tryptic peptides that confound simplistic analysis. While the simplest model of tryptic peptide identity has been well grounded with respect to random expectation, the more complex assumptions associated with phosphopeptides remain to be examined alongside random spectra [31, 38, 54–56] and so are more speculative [64]. Examining the gene symbol intensity across all twelve disease and controls clinical sample sets by box plots and two-way ANOVA was a direct means to compare across all other diseases and controls to look for proteins specific to ICU-Sepsis. Many of the proteins that show increased observation in ICU-Sepsis independently showed greater log_10_ intensity values that was consistent with true-positive variation between ICU-Sepsis and ICU Control. The FN1, ITIH3, SAA1 and other peptides observed were consistent with previous claims of utility for these proteins as sepsis biomarkers [68, 69]. Ultimately, analysis of peptides across all treatments will be required to extract all of the information from such as large dataset and will require large and automated computations.

### Agreement with previous genetic and biochemical experiments

SAA1 has been implicated in many different diseases including sepsis [1] and the intact full length protein is unlikely to serve as a specific biomarker [58]. Tryptic peptides cleaved in plasma from SAA1 showed a higher observation frequency and/or mean log_10_ intensity in sepsis. The SAA1 tryptic peptide D/PNHFRPAGLPEKY, was cleaved from the COOH terminus of SAA1 and was specific to ICU and ICU-Sepsis patients similar to that observed in patients with the auto inflammatory disorder Kawasaki’s disease [70]. The location of the most frequent SAA1 cleavage site COOH terminal of lysine (K108) was also the most hydrophilic point of SAA1 as calculated by the method of Kyte and Doolittle [71]. Thus, ICU-Sepsis showed cleavage on the COOH side of the previously established Cystatin C binding site [72] and elsewhere more frequently than any other disease or control treatment. Moreover, the D/PNHFRPAGLPEKY peptide was not observed in ice cold normal samples shown herein or in similar samples warmed to room temperature [39] and so COOH cleavage of SAA1 does not apparently result from poor sample handling. Furthermore, SAA1 cleavages on the NH2 terminal side of the Cystatin binding site were observed in ICU-Sepsis but not ICU Control. The sepsis-specific peptides apparently result from the ex vivo action of proteases from septic patients that are secreted or activated as a part of the host response to infection. It should be possible to specifically compare and confirm the disease specific expression of peptides and/or parent proteins by automatic targeted proteomics [40] after extraction of peptides [43], or after collection of the parent protein over the best partition chromatography resin followed by exogenous tryptic digestion [41] to test the discovery on a larger set of samples. For example, C4B peptides discovered by random and independent sampling were shown to be a marker of sample degradation by automatic targeted assays [39, 40, 65]. Automatic targeted analysis of peptides was an independent analysis that provided relative quantification to rapidly confirm the potential utility of C4B peptide as a marker of sample degradation [40]. Subsequently, the best performing peptides may be absolutely quantified by external or internal-isotopic standards to provide absolute quantification. Thus, by monitoring the processing of SAA1 in EDTA plasma by LC–ESI–MS/MS it might be possible to detect and resolve ICU-Sepsis patients from the background population of ICU.

## Conclusion

It was possible to discover peptides and/or proteins that showed variation specific to sepsis versus other diseases, or normal plasma samples, from many institutions using simple, disposable sample preparation, common bench-top instrumentation, and generic computation. The results of the step-wise organic extraction of peptides [42] provided for the enrichment of endogenous tryptic peptides with high signal to noise for random sampling [40] across disease and normal treatments. A large amount of MS and MS/MS spectra data from multiple diseases, controls and institutions may be collected by random and independent sampling with a set of robust and sensitive linear quadrupole ion traps and fit to peptides using X!TANDEM or SEQUEST and stored, related and statistically analyzed in 64 bit SQL Server/R. Analyzing the resulting database by Chi square and ANOVA with SQL Server R identified proteins and peptides specific to ICU-Sepsis versus other diseases. The LC–ESI–MS/MS of plasma endogenous tryptic peptides identified many blood proteins and/or peptides elevated in ICU-Sepsis versus ICU Control that were previously associated with the innate immune response. Cleavage of the SAA1 protein to release peptides from the COOH terminal and elsewhere was more frequent in sepsis compared to all other diseases and controls. SAA1 peptides discovered by random and independent sampling of test samples might be confirmed by automatic targeted LC–ESI–MS/MS [39, 40, 65] from a larger cohort of independent samples.

## Supplementary information

**Additional file 1: Table S1.** The tryptic peptides with Chi square FDR q-values.

**Additional file 2: Table S2.** The tryptic phosphopeptides with Chi square FDR q-values.

**Additional file 3: Table S3.** Gene Ontology distributions of the Chi square data from corrected frequency that differ by at least 10 observations and a Chi square χ^2^ > 9. Network statistics: number of nodes, 484; number of edges, 1147; average node degree, 4.74; avg. local clustering coefficient, 0.324; expected number of edges, 850; PPI enrichment p-value: < 1.0e−16.

## Data Availability

The raw data is provided in companion publication and its additional files.

## Abbreviations

TRYP: Fully tryptic
TRYP STYP: Fully tryptic and/or S, T or Y tryptic phosphopeptide
ICU: Intensive Care Unit
